# The influence mechanism of academic involution behavior among Chinese college students: a moderated mediation analysis based on the JD-R model

**DOI:** 10.3389/fpsyg.2026.1729314

**Published:** 2026-03-06

**Authors:** Xiangwen Ji, Hanqiang Li

**Affiliations:** Faculty of Social Sciences and Liberal Arts, UCSI University, Kuala Lumpur, Malaysia

**Keywords:** academic anxiety, academic involution behavior, job demands–resources (JD–R) model, perceived employability, upward social comparison

## Abstract

**Introduction:**

Against the backdrop of increasingly intense employment competition, Chinese university students are experiencing growing pressure to enhance their competitiveness. This study examined the relationship between perceived employability and academic involution behavior, focusing on the mediating role of upward social comparison and the moderating role of academic anxiety within the framework of the Job Demands–Resources (JD–R) model.

**Methods:**

A cross-sectional survey was conducted among 498 Chinese undergraduates using standardized questionnaires assessing perceived employability, upward social comparison, academic anxiety, and academic involution behavior. Structural equation modeling and bootstrapping procedures were employed to test a moderated mediation model.

**Results:**

Perceived employability was positively associated with academic involution behavior, and this relationship was partially mediated by upward social comparison. The indirect effect accounted for approximately 11% of the total effect. Academic anxiety significantly moderated the path from perceived employability to upward social comparison, such that the mediating effect was stronger under higher levels of academic anxiety.

**Discussion:**

The findings extend the JD–R model to academic settings by demonstrating how perceived employability may promote competitive academic behaviors through social comparison processes. The moderating role of academic anxiety highlights the conditional nature of this mechanism. These results provide theoretical insight into student motivational dynamics and offer practical implications for reducing maladaptive academic competition in higher education contexts.

## 1 Research background

In recent years, employment instability has emerged as a global challenge with wide-ranging implications across multiple domains of sociological inquiry (Kalleberg, 2009). China has undergone profound economic transformation, accompanied by a substantial expansion of higher education, with both university enrollment and graduation rates increasing markedly. Between 2000 and 2024, participation in higher education reached a historical high (Shi, 2024). Against this backdrop, involution has gradually become a high-frequency term in contemporary Chinese society, particularly among university populations.

The concept of involution originates from an academic tradition in anthropology, where it was used to describe a structural dilemma characterized by high input but low output (Fearnley and Sun, 2025). Within the university student population, involution primarily manifests as individuals continuously intensifying their academic effort under conditions of limited educational and developmental resources in order to maintain or enhance their relative competitive position, yet failing to obtain commensurate developmental returns (Zhang et al., 2025). In the Chinese context, academic involution behaviors among university students are typically reflected in excessive time devoted to studying, inefficient forms of competitive effort, the reinforcement of grade- and ranking-oriented behaviors, and heightened anxiety related to failure and falling behind peers (Liu A. et al., 2024). Such behaviors do not necessarily lead to substantive academic gains; instead, they may undermine intrinsic learning motivation and exert detrimental effects on students’ mental health and long-term development (Lai and Ng, 2026).

The emergence of this phenomenon is closely intertwined with the broader macro-social environment. The prevalence of overeducation has also prompted scholars to question the validity of certain core assumptions and predictions of human capital theory (McGuinness, 2006). In many cases, the scarcity of social resources serves as a key trigger for involutionary dynamics. In pursuing limited opportunities, such as internships or jobs, students often face fierce competition and escalating pressure (Wang et al., 2024). Academic involution has been conceptualized as a competitive phenomenon whose defining feature lies in students’ continuous escalation of effort to compete for scarce educational resources, a process that is frequently accompanied by diminishing marginal returns, reflected in a persistent decline in the effort–reward ratio (He and Liu, 2025). As the level of academic involution among university students increases, academic pressure correspondingly intensifies, leading to greater psychological depletion and internal strain (Liu et al., 2025). Han and Chi (2012) further noted that involutionary behaviors among university students give rise to anxiety across learning, daily life, and social interactions, posing serious threats to both mental and physical health.

Existing empirical research indicates a significant association between perceived employability and academic involution behaviors among university students; however, most studies have tended to focus on single-variable pathways (Ma and Bennett, 2021). For example, some research has examined the impact of perceived employability on academic performance (Mohamad et al., 2025), while other studies have explored the effects of academic involution on learning motivation and academic stress (Liu C. et al., 2024). Relatively few studies have sought to explain how perceived employability is transformed into academic pressure and involutionary behaviors within a systematic theoretical framework, such as the Job Demands–Resources (JD–R) model. Examining the relationship between academic involution and perceived employability through the lens of the JD–R model not only helps address theoretical gaps in the existing literature, but also provides a more integrated and comprehensive perspective for understanding students’ academic behaviors and psychological states.

## 2 Literature review

### 2.1 Precarious employment

Precarious employment refers to forms of work characterized by job insecurity, low wages, limited social protection, and the absence of long-term contracts (Jaydarifard et al., 2023). Unlike traditional forms of stable employment, precarious employment exposes individuals to uncertain labor market outcomes and heightened risks of income volatility (Alon, 2023). This perspective emphasizes how structural features of the labor market generate differentiation and inequality in employment opportunities across social groups, thereby exacerbating broader patterns of social inequality (Gebel and Gundert, 2023). From this standpoint, precarious employment is not merely an economic condition, but also a structural risk factor that shapes individual behavior and psychosocial outcomes (Irvine and Rose, 2024).

A growing body of empirical research has documented the wide-ranging consequences of precarious employment across multiple key social domains. Precarious work constrains stable career development, intensifies competition for a limited number of secure jobs, and thereby reinforces labor market stratification (Xu et al., 2024). Research in occupational health psychology has consistently shown that employment insecurity is associated with higher levels of stress, anxiety, and other adverse mental health outcomes (Kvart et al., 2021). Individuals operating in precarious work environments experience greater performance pressure and heightened uncertainty about their future prospects (Alon, 2023). Precarious employment has been linked to reduced social mobility, as constrained income and unequal access to career advancement opportunities limit individuals’ long-term chances of improving their socioeconomic status (Bosmans, 2023). In the family sector, relevant research indicates that employment instability can lead to delays or interruptions in family formation decisions due to economic insecurity and uncertainty about survival (Mugiyama, 2025). In the educational domain, students’ perceptions of a precarious labor market environment have been shown to influence their educational trajectories, including major selection, patterns of learning engagement, and expectations for the future (Zhong and Xu, 2023).

Given these multifaceted effects, precarious employment constitutes a structural context that shapes students’ perceptions of employability and their learning behaviors (Sánchez-Queija et al., 2023). In the present study, precarious employment is conceptualized as a contextual condition that heightens students’ concerns about employability and elicits compensatory learning behaviors under conditions of competitive uncertainty. This conceptualization situates the study’s hypotheses within established sociological and psychological frameworks and provides a coherent foundation for the proposed relationships.

### 2.2 The theoretical framework of the JD–R model

The Job Demands–Resources (JD–R) model was originally proposed by Demerouti et al. (2001) and subsequently extended by Bakker and Demerouti (2007, 2017). It has become one of the most influential theoretical frameworks for explaining how individuals cope with stress and maintain motivation in both work and learning contexts. Lesener et al. (2020) argued that university study activities can essentially be regarded as a form of work process, in which students are required to deal with heavy coursework, time constraints, and competitive pressures, while continuously allocating and investing limited temporal, energetic, and psychological resources.

The core premise of the JD–R model is that individuals’ behavioral and psychological outcomes are shaped by the dynamic balance between job demands and job resources. At the heart of the JD-R model, excessive demand depletes an individual’s energy, leading to burnout and job burnout, while resources can buffer the negative effects of demand and stimulate motivation and active participation (Bakker et al., 2005). When individuals exhibit a high degree of self–goal congruence, their intrinsic motivation is more effectively activated, resulting in greater persistence and engagement in goal pursuit (Bakker et al., 2014).

Hong et al. (2022) argue that when students compare themselves to better-performing peers during the learning process, they may be motivated to improve themselves, which can trigger self-regulation to compensate for perceived deficiencies. In the second core proposition of the JD-R model, perceived employability can be used as both an intrinsic and extrinsic incentive resource. It operates as an intrinsic resource insofar as it facilitates competence development, learning accumulation, and career advancement, and as an extrinsic resource insofar as it provides conditions and support for achieving work- and study-related goals (Bakker and Demerouti, 2007).

In higher education contexts, an academic involutionary climate and employment pressure can be conceptualized as academic demands, characterized by intense competition, scarce resources, and strong performance orientation (Diabat and Bellibas, 2025). These conditions often elicit heightened academic stress and anxiety among students. Bakker et al. (2023) argued that excessively high demands and pressure may trigger maladaptive self-regulatory processes, manifested in impairments in cognitive and behavioral functioning. Under high workload conditions, resource allocation must be balanced across multiple concurrent tasks or distractions; once capacity limits are exceeded, monitoring failures and delayed responses are likely to occur (Hockey, 1997). Applying the JD–R model to the study of university students’ employability perceptions and academic behaviors can therefore help integrate previously fragmented findings and provide a coherent explanation for the complex relationships among perceived employability, academic anxiety, upward social comparison, and academic involution behavior.

### 2.3 Perceived employability

Perceived employability refers to individuals’ perceptions of whether they possess the skills and other attributes required to obtain and maintain their desired employment (Rothwell and Arnold, 2007). In research on university students’ job preferences, core dimensions commonly assessed include long-term job stability, economic returns, intrinsic interest and personal development, and occupational prestige (Culbertson et al., 2009). Job resources are understood to exist at multiple levels. At the organizational level, they are primarily reflected in salary, career development opportunities, and job security; at the interpersonal and social level, they manifest as supervisory support, peer assistance, and a positive team climate; at the work organization level, key resources include role clarity and employees’ participation in decision-making processes; and at the task level, resources encompass skill variety, task identity, task significance, autonomy, and timely and effective performance feedback (Bakker and Demerouti, 2007).

Within the contemporary employment context, perceived employability places greater emphasis on students’ subjective experiences and psychological evaluations, and is readily influenced by multiple factors, including individual competencies, educational background, social networks, and external labor market conditions (Álvarez-González et al., 2017). Existing literature suggests that university students’ perceived employability tends to increase alongside the accumulation of knowledge acquired during their developmental trajectory, which is consistent with progressive gains in self-confidence, experience, and self-esteem (Donald et al., 2018). When perceived employability is higher, students are more likely to invest greater time and effort in learning activities, thereby enhancing learning engagement and effort levels (Ma and Bennett, 2021).

When the job demand-resource model is applied to a university setting, resources related to learning and career development, such as institutional reputation, course quality, practical training opportunities, and career support services, can be considered equivalent functions of job resources (Ergün and Şeşen, 2021). This perspective supports the inclusion of higher education learning and career development resources within the resource domain of the JD–R model to explain the formation and development of perceived employability.

### 2.4 Upward social comparison (USC)

Social comparison theory was originally proposed by Festinger (1954). Within this theoretical framework, upward social comparison (USC) constitutes a core concept and refers to the process by which individuals compare themselves with others who perform better or are more successful in a given domain (Buunk and Gibbons, 2007). Upward social comparison exhibits a dual nature. When the comparison target is perceived as an attainable role model, USC can stimulate learning motivation and self-improvement orientation; however, when the perceived gap is excessively large or accompanied by negative emotional experiences, USC may elicit feelings of frustration and psychological exhaustion, thereby inhibiting positive behavioral engagement (Suls et al., 2002). Qualities closely related to future orientation, such as proactively developing new skills and being keen on emerging opportunities, can continuously enhance an individual’s perception of their own employability (Savickas and Porfeli, 2012; Wittekind et al., 2010).

When students compare themselves to better-performing peers during learning activities, they typically experience a greater motivation to improve themselves and then attempt to compensate for perceived deficiencies through self-regulation or behavioral adjustments (Hong et al., 2022). Within the JD–R model framework, USC can amplify students’ psychological strain. In contexts characterized by employment pressure and frequent peer academic comparisons, students with abundant personal resources (such as self-efficacy, psychological resilience, and social support) are more likely to see the strengths of others as opportunities for learning and growth rather than threats (Zeijen et al., 2024). Nevertheless, frequent comparison increases students’ psychological burden and depletes their cognitive and emotional resources (Buunk and Gibbons, 2007). This pathway largely originates from negative emotional experiences triggered by the comparison process and a weakened sense of subjective control, which may drive excessive yet inefficient competition and ultimately evolve into maladaptive competitive behaviors (Qi et al., 2024).

### 2.5 Academic involution behavior

The term involution was originally introduced by the anthropologist Goldenweiser (1937) to describe a cultural pattern that, after reaching a certain form, is unable to transform into new forms and instead becomes increasingly complex internally. Subsequently, Geertz (1963) applied this concept in his seminal work on Indonesian agriculture, arguing that under conditions where land and resources cannot expand outward, labor input may continue to intensify while output growth stagnates, resulting in a structural dilemma characterized by high input and low output. In its original formulation, involution referred to a state of structural stagnation marked by increasing internal differentiation without substantive development (Fearnley and Sun, 2025). This early usage operated at the macro level and was not intended to describe individual psychological or behavioral processes (Xu et al., 2025). Over time, the concept of involution has extended beyond its anthropological origins and has been more widely adopted in sociological discourse to describe increasingly intensified forms of competition within constrained structural environments (Wang et al., 2022). In this context, the term “involution” refers to a situation in which, in a social structure with limited opportunities, group members continuously increase their efforts to gain a relative advantage, but the overall gains fail to increase accordingly (Yang et al., 2025). Such lines of research emphasize the interaction between structural constraints and competitive dynamics, rather than focusing solely on individual motivation (Ma X. et al., 2025).

From a macro-level perspective, China’s higher education system is currently undergoing a widespread transition from expansion in scale to competition centered on quality (Jiang and Lui, 2024). As the massification of higher education continues, the growth of high-quality employment opportunities has lagged behind (He et al., 2020). Within this context, academic performance has increasingly been embedded in a framework of intensive comparison and ranking, where grade rankings, grade point average (GPA), scholarship eligibility and eligibility for further study have become important criteria for evaluating students’ worth. This structural imbalance has led university students to widely perceive intense competitive pressure in both their academic and career development processes (Gobena, 2024).

Existing research indicates that in highly competitive and outcome-oriented higher education environments, students’ learning motivation is increasingly shaped by external goals, such as employability and performance indicators (Ferrer et al., 2022). Students often exhibit excessive investment of study time, including prolonged study hours, reduced rest periods, and the sacrifice of opportunities for physical and psychological recovery, in an effort to maintain a competitive advantage (Benítez-Agudelo et al., 2025). Such high-intensity investment does not necessarily translate into improvements in learning efficiency or quality; instead, it may result in attentional decline and learning fatigue (Lin, 2021). Academic involution is also closely associated with heightened anxiety related to failure and relative underperformance. Studies have shown that in competitive academic environments, students remain highly vigilant toward grade fluctuations, ranking declines, and uncertainty regarding future development (Benítez-Agudelo et al., 2025). This persistent state of psychological tension not only elevates levels of academic stress, but may also give rise to anxiety, psychological depletion, and emotional exhaustion, thereby further undermining learning experiences and mental health (Cabras et al., 2024). In such intensely competitive climates, students are driven to continually invest additional effort to sustain their competitive position, while simultaneously increasing the risk of academic strain and compromised mental health, ultimately hindering balanced development (Wang et al., 2024).

According to the core assumptions of the JD–R model, academic involution can be understood as a process in which individuals, under conditions of limited resources, are compelled into continuous competition and self-depletion, leading to a persistent decline in the effort–reward ratio over time (Ling and Li, 2022). Academic involution is therefore not merely a consequence of external academic demands, but also reflects individuals’ psychological depletion and behavioral choices under resource-scarce conditions (Wen and Jin, 2025). This perspective provides a theoretical foundation for examining the interactions among perceived employability, upward social comparison, and academic anxiety (Ma J. et al., 2025).

### 2.6 Academic anxiety

Academic anxiety, as a typical achievement-related emotion, is widely experienced by students throughout the learning process and is regarded as a common psychological response during academic development (Zhou et al., 2025). It has received extensive attention in the field of educational psychology. Pekrun (2006), in his Control-Value Theory, posited that academic anxiety is a negative emotional response arising when individuals perceive a task outcome as highly valuable but feel insufficient control over it. Academic anxiety not only affects students’ learning motivation and cognitive processing but can also further undermine academic performance. Zeidner (1998), from the perspective of test anxiety, systematically elaborated on the broad impact of academic anxiety on students’ psychological states and academic outcomes, emphasizing that excessive anxiety may lead to attentional distraction, reduced learning efficiency, and compromised physical and mental health.

From the perspective of the JD–R model, academic anxiety can be conceptualized as a typical negative emotional response that students exhibit when coping with learning demands and academic pressure. When students perceive high employment-related pressure, those with lower anxiety are more likely to channel this pressure into goal-directed effort. Research among Finnish university students indicates that when students anticipate a large gap between their abilities and the actual requirements of the labor market, they tend to hold more pessimistic perceptions of their employability, even when their actual abilities may not be low (Räty et al., 2019). Gao (2023) suggested that students with higher anxiety levels are more prone to experience intense burnout and decreased efficiency, whereas students with lower anxiety levels are more likely to transform pressure into relatively sustained and stable learning effort. Bandura (1997) argued that individuals with high self-efficacy are more inclined to set clear goals, adopt planned strategies, and persist in their execution, making it more likely that they will convert pressure into effective effort under stressful conditions.

### 2.7 Research hypotheses

**Figure 1:**
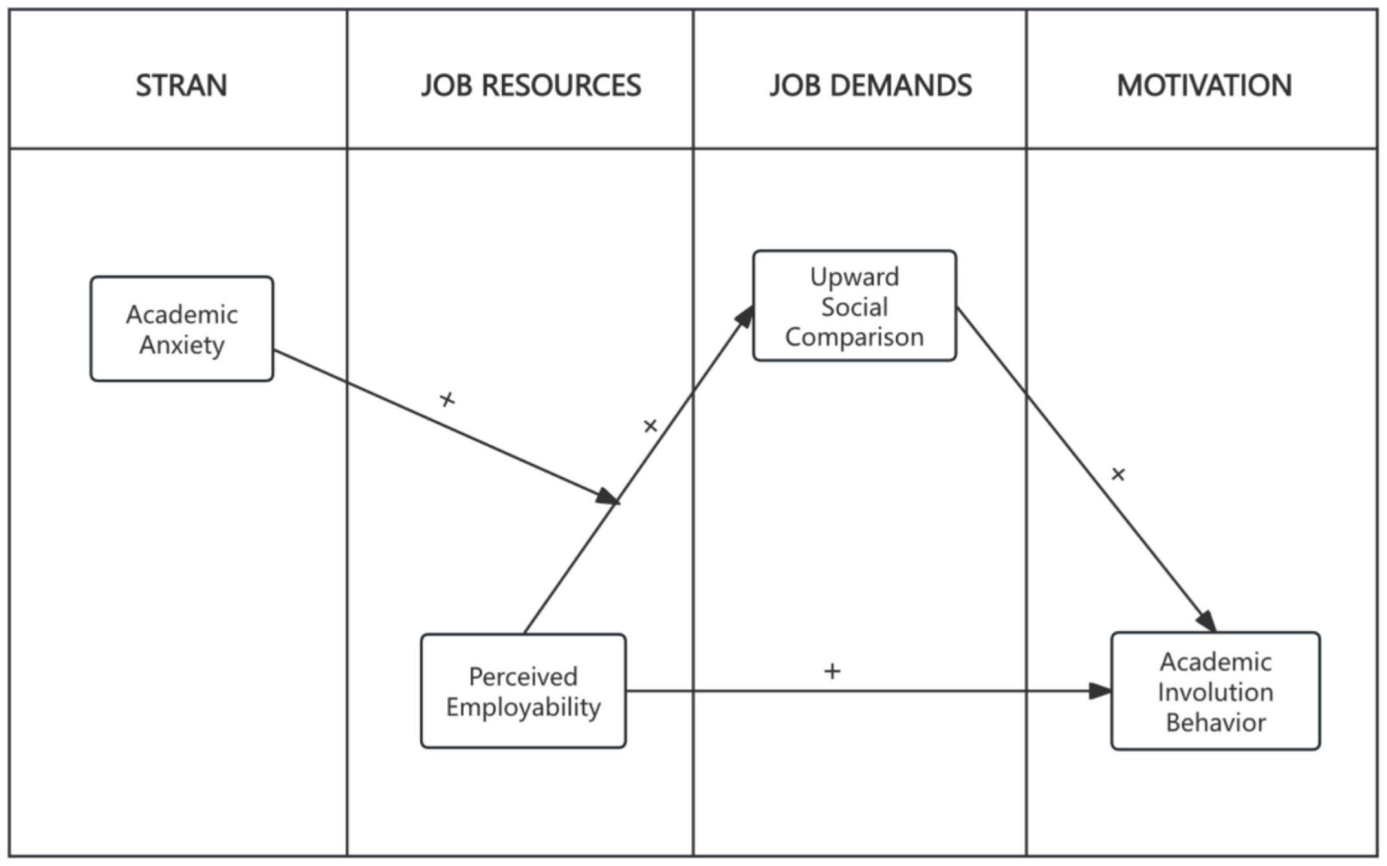
Conceptual framework of the hypothesized mediation model with contextual moderation.

Based on the JD–R model, this study examines the relationships among perceived employability, upward social comparison, academic involution behavior, and academic anxiety. Accordingly, the following research hypotheses are proposed (see Figure 1):

According to the Conservation of Resources (COR) theory (Hobfoll, 2001), individuals strive to acquire, retain, and protect the things they value, including material, social, personal, or energetic resources. Job resources are defined as the physical, psychological, social, or organizational aspects of work that help achieve work goals or facilitate personal growth, learning, and development (Arnold, 2007). Students’ perceived employability can be regarded as an accessible job resource. Available resources can shape individuals’ perceptions of their goals, thereby influencing the amount of effort they invest (Schmid, 2022). A heightened focus on employability may implicitly increase students’ psychological and behavioral burden, motivating them to devote more time and energy to maintaining a competitive advantage (Ma and Bennett, 2021). When individuals operate under resource-constrained conditions, such passive competitive behavior can easily lead to an imbalance between input and output, evolving into a low-efficiency, self-draining competitive state (Demerouti et al., 2001). Based on this, we propose the following hypothesis:

*H1*: Perceived employability positively predicts academic involution behavior.

According to social comparison theory, individuals continually evaluate their relative standing compared to others in competitive environments (Bosch and Wilbert, 2023). High perceived employability does not operate in isolation; it interacts with self-perceptions and others’ expectations, potentially enhancing the tendency toward upward social comparison (Räty et al., 2019). Previous research has shown that concerns about one’s employability are closely associated with more frequent comparative behaviors in educational settings (Rothwell et al., 2008). Therefore, we propose the following hypothesis:

*H2*: Perceived employability positively predicts upward social comparison.

Social comparison theory posits that frequent upward comparisons intensify individuals’ perception of performance gaps and reinforce competitive behaviors (Sung et al., 2024). In academic contexts, this heightened comparison process may lead to overengagement, repetitive effort, and diminishing marginal returns (Räty et al., 2020). Empirical evidence indicates that an excessive tendency for comparison is associated with maladaptive patterns of academic engagement (Vazquez et al., 2023). Therefore, we propose the following hypothesis:

*H3*: Upward social comparison positively predicts academic involution behavior.

In highly competitive and outcome-oriented higher education environments, academic anxiety increases students’ attention to peers’ performance and relative standing, thereby enhancing social comparison motivation (Schlechter et al., 2025). Although perceived employability, as a personal resource, helps individuals form judgments about their own competitive capabilities, under conditions of high academic anxiety, students are more likely to engage in upward comparisons with higher-performing peers to assess whether their resources are sufficient to cope with future uncertainties (Huynh et al., 2024). Therefore, we propose the following hypothesis:

*H4*: Academic anxiety positively moderates the predictive relationship between perceived employability and upward social comparison.

## 3 Research method

### 3.1 Research design

This study employed a cross-sectional survey design to collect quantitative data. The collected data were analyzed using RStudio (Version: 2026.01.0 + 392) to examine the mechanisms and robustness of the relationships among the study variables.

### 3.2 Preliminary questionnaire design

The questionnaire used in this study consisted of four sections, with all scale items measured on a standard five-point Likert scale. Items were retained based on factor loadings of at least 0.50, theoretical relevance, and satisfactory discriminative power, while items with high cross-loadings or low reliability were removed to ensure the structural validity and reliability of the scales. The demographic section collected participants’ gender, age, field of study, and institutional level. The scale section included four separate scales:

#### 3.2.1 Self-perceived employability scale

This scale was revised by Rothwell et al. (2008) and originally contained 16 items across four fundamental dimensions: self-belief (e.g., “My academic performance is excellent”), university reputation (e.g., “My university is highly regarded in my field of study”), intrinsic factors of the job seeker (e.g., “The skills and abilities I possess are exactly what employers value”), and the current state of the external labor market (e.g., “There is high overall demand for graduates in today’s society”). These dimensions interact to form eight core sub-dimensions: academic effort and performance, perceived university brand strength, reputation of the university within the field, professional status and credibility, professional demand in the labor market, perception of labor market conditions, perceived market opportunities, and confidence in personal skills. To reduce redundancy, prevent respondent fatigue, minimize careless responses, and ensure measurement comprehensiveness, eight core items (one per sub-dimension) were selected. During exploratory factor analysis (EFA), items Q1 and Q4 had factor loadings of 0.32 and 0.41, respectively, which are below the recommended threshold of 0.50; these items were therefore removed. Based on the factor loading results, the final version of the scale retained six items.

#### 3.2.2 Academic involution scale for university students

This scale was revised by Wang et al. (2024) and originally contained 16 items across three dimensions: academic behaviors (e.g., “I attend private tutoring sessions to improve myself so as not to fall behind others,“7 items), social activities (e.g., “Even if reluctantly, I join various student organizations to ensure my overall evaluation does not fall behind others,” 5 items), and social interactions (e.g., “I maintain good relationships with classmates to avoid losing in evaluations,” 4 items). For measuring academic involution behavior in this study, and considering the characteristics of the participants and learning context, we selected the dimension most relevant to students’ learning behaviors, consisting of seven items. During exploratory factor analysis (EFA), item Q13 had a standardized factor loading of 0.30, which is below the recommended threshold of 0.50, and was therefore removed. Based on the factor loading results, the final version of the scale retained six items.

#### 3.2.3 Social comparison orientation scale

This scale was revised by Rose et al. (2024) and originally included 23 items across three subscales: the non-directional comparison subscale (e.g., “I often compare myself with others in terms of achievements in life,” 11 items), the upward comparison subscale (e.g., “In personal life, I sometimes compare myself with people who are better off than me,” 6 items), and the downward comparison subscale (e.g., “In personal life, I sometimes compare myself with people who are worse off than me,” 6 items). Among these, the upward comparison subscale measures individuals’ proactive tendency to compare themselves with those who are superior, which closely aligns with the core construct of this study. Therefore, this subscale was selected as the primary measurement tool. Based on factor loading results, all six items of the upward comparison subscale were retained.

#### 3.2.4 Academic anxiety scale

This scale was revised by Cassady et al. (2019) and originally included 11 items across three dimensions: general neuroticism (e.g., “There are always some things at school that make me feel scared”), academic anxiety (e.g., “I often worry that I have not completed my assignments correctly”), and cognitive test anxiety (e.g., “I find it difficult to manage various school-related tasks”). During exploratory factor analysis (EFA), item Q23 had a standardized factor loading of 0.38, below the recommended threshold of 0.50, and was therefore removed. Based on factor loading results, the final version of the scale retained 10 items.

### 3.3 Reliability and validity testing of the scales

After revising the original scales, 520 questionnaires were randomly distributed to Chinese university students via an online platform, and 498 valid responses were collected, yielding an effective response rate of 95.8%. Reliability analysis was first conducted for the complete structural model, which included four dimensions: perceived employability (6 items, Cronbach’s *α* = 0.82), academic involution behavior (6 items, Cronbach’s α = 0.78), upward social comparison (6 items, Cronbach’s α = 0.82), and academic anxiety (10 items, Cronbach’s α = 0.91). The overall scale demonstrated a Cronbach’s α of 0.77. Methodological research indicates that Cronbach’s α values between 0.70 and 0.80 are generally considered acceptable in social science research (Taber, 2018; Tavakol and Dennick, 2011). The Kaiser–Meyer–Olkin (KMO) measure was 0.903, and Bartlett’s test of sphericity was significant (*p* < 0.001), confirming good internal consistency and suitability for confirmatory factor analysis (CFA). The scales also exhibited adequate convergent validity, with an average variance extracted (AVE) of 0.43 and composite reliability (CR) of 0.81. Although AVE is slightly below the recommended threshold of 0.50, the CR exceeds the recommended cutoff (CR > 0.70), and previous research suggests that if CR ≥ 0.70 and standardized factor loadings (*λ* > 0.50) are satisfactory, a construct can still be considered to have acceptable convergent validity even when AVE is slightly lower (Cheung et al., 2024). This indicates that the scales possess internal consistency and reliability.

To examine the structural validity of the scales, both exploratory factor analysis (EFA) and confirmatory factor analysis (CFA) were conducted. In the EFA, the Kaiser–Meyer–Olkin (KMO) measure was 0.895 and Bartlett’s test of sphericity was significant (p < 0.001), indicating that the data were suitable for factor analysis. Principal axis factoring was employed with the number of factors fixed at four, and an oblique rotation with factor alignment was applied. After removing items with factor loadings below 0.45, EFA was re-conducted on the retained items. The results showed that all standardized factor loadings exceeded 0.45, and the cumulative variance explained was 54.29%, suggesting that the final factor structure demonstrated satisfactory structural validity (see Table 1).

**Table 1:** Exploratory factor analysis of scale.

Factor	Item	Primary factor	h^2^	% of variance	Cumulative %	Processing results
PE	Q1	0.32	0.31	25.21	25.21	Factor loading <0.45, removed
PE	Q2	0.77	0.61	14.78	39.99	Preserve results
PE	Q3	0.73	0.68	6.23	46.22	Preserve results
PE	Q4	0.41	0.33	5.54	51.75	Factor loading < 0.45, removed
PE	Q5	0.64	0.39	3.47	55.22	Preserve results
PE	Q6	0.73	0.53	3.33	58.55	Preserve results
PE	Q7	0.57	0.52	2.85	61.40	Preserve results
PE	Q8	0.50	0.59	2.50	63.90	Preserve results
AIB	Q9	0.50	0.31	2.50	66.37	Preserve results
AIB	Q10	0.50	0.33	2.36	68.73	Preserve results
AIB	Q11	0.55	0.34	2.34	71.07	Preserve results
AIB	Q12	0.63	0.35	2.06	73.13	Preserve results
AIB	Q13	0.30	0.32	2.03	75.16	Factor loading < 0.45, removed
AIB	Q14	0.72	0.49	2.00	77.16	Preserve results
AIB	Q15	0.79	0.59	1.94	79.09	Preserve results
USC	Q16	0.61	0.43	1.85	80.94	Preserve results
USC	Q17	0.67	0.44	1.78	82.73	Preserve results
USC	Q18	0.78	0.60	1.65	84.37	Preserve results
USC	Q19	0.62	0.48	1.55	85.92	Preserve results
USC	Q20	0.55	0.35	1.47	87.40	Preserve results
USC	Q21	0.73	0.51	1.43	88.83	Preserve results
AA	Q22	0.56	0.53	1.35	90.18	Preserve results
AA	Q23	0.38	0.43	1.29	91.48	Factor loading <0.45, removed
AA	Q24	0.60	0.66	1.20	92.68	Preserve results
AA	Q25	0.48	0.64	1.13	93.81	Preserve results
AA	Q26	0.88	0.68	1.08	94.89	Preserve results
AA	Q27	0.71	0.52	1.01	95.89	Preserve results
AA	Q28	0.82	0.64	0.93	96.83	Preserve results
AA	Q29	0.80	0.62	0.89	97.72	Preserve results
AA	Q30	0.50	0.53	0.85	98.57	Preserve results
AA	Q31	0.75	0.53	0.76	99.32	Preserve results
AA	Q32	0.77	0.57	0.68	100.00	Preserve results

Subsequently, confirmatory factor analysis (CFA) was performed using RStudio. The results indicated a good model fit (*χ*^2^/df = 2.14, *p* < 0.001, CFI = 0.913, TLI = 0.904, RMSEA = 0.048, SRMR = 0.064). Taken together, these findings confirm that the revised measurement instruments exhibit adequate structural validity within the target population and cultural context, supporting their suitability for subsequent analyses.

### 3.4 Ethical considerations and informed consent

This study strictly adhered to ethical standards. All research procedures were reviewed and approved by the Ethics Committee of the School of Psychology and Educational Sciences at Zaozhuang University. Prior to questionnaire distribution, participants were informed of the purpose of the study, the voluntary nature of participation, the anonymity of responses, and the confidentiality of all collected data. All participants took part voluntarily and completed the questionnaire only after signing an informed consent form. The study posed no potential risks to participants, and all collected data were used exclusively for academic research purposes, without any commercial or non-research applications. Throughout the entire research process, participants’ rights to privacy were fully protected, and strict measures were taken to ensure data anonymity, confidentiality, and security.

## 4 Research results

### 4.1 Questionnaire distribution and demographic information

A random sampling strategy was employed. Data were collected via the Credamo online survey platform,^1^ which randomly distributed the questionnaire to currently enrolled university students across China. Detailed demographic information of the sample is presented in Table 2.

**Table 2:** Demographic information of the sample.

Variable	Category	Frequency (n)	Percentage (%)
Gender	Male	124	24.9%
	Female	374	75.1%
Age	<19	2	0.4%
	19–21	302	60.6%
	22–24	194	39%
School level	Ordinary undergraduate colleges	350	70.3%
	Double First-Class University	148	29.7%
Academic major	Teacher Education	67	13.5%
	Science and Engineering	178	35.7%
	Literature and History	62	12.4%
	Medical Sciences	33	6.63%
	Business and Management	141	28.3%
	Others	17	3.41%

### 4.2 Pearson correlation analysis

To examine the linear relationships among the variables, this study conducted a Pearson correlation analysis. The results indicated that Perceived Employability (PE), Academic.

Involution Behavior (AIB), Upward Social Comparison (USC), and Academic Anxiety (AA) were significantly correlated with one another. Detailed results of the analysis are presented in Table 3.

**Table 3:** Means, Standard Deviations, and Correlations Among Study Variables

Variable	M±SD	1	2	3	4
1. PE	3.08±.85	1			
2. AIB	3.59±.72	.439**	1		
3. USC	3.69±.73	.180**	.324**	1	
4. AA	2.60±.88	−.476**	−.197**	.122**	1

*p < 0.05, **p < 0.01, ***p < 0.001.

These findings provide a theoretical foundation for the subsequent structural equation modeling.

(SEM) analysis.

### 4.3 Model construction and mediation effect testing

In this study, the structural model was tested using the lavaan package in R, with perceived employability as the independent variable, academic involution behavior as the dependent variable, and upward social comparison as the mediating variable. Maximum likelihood estimation (ML) was employed in combination with robust standard errors (sandwich estimator) to enhance the robustness of the estimates. All model fit indices indicated good fit (*χ*^2^/df = 2.14, *p* < 0.001; CFI = 0.913; TLI = 0.904; RMSEA = 0.048; SRMR = 0.064).

To test the mediation effect, this study employed the bias-corrected percentile Bootstrap method, using 5,000 resamples to calculate the 95% confidence intervals (CIs) of the indirect effect. As shown in Table 4, the 95% CIs for all direct effect paths did not include zero, and the *p*-values for three of the paths were less than 0.05, indicating that the direct effects were statistically significant. The total effect was 0.574, and the indirect effect was 0.063, accounting for 11% of the total effect, suggesting that upward social comparison partially mediates the relationship between perceived employability and academic involution behavior. Cano-Corres et al. (2012) emphasized that effect size is an important complement to tests of statistical significance, as it helps to evaluate the practical meaningfulness of differences. Even when p-values are significant, the practical importance of the effect should be assessed. The path analysis for the indirect effect from perceived employability to academic involution behavior showed a 95% Bootstrap confidence interval of 0.498 to 0.922, which does not include zero, and a p-value less than 0.05. Although the effect size in this study was relatively small, the bias-corrected and percentile Bootstrap confidence intervals allow for accurate detection and interpretation of the estimate, even when the effect is modest (Walters, 2019). Therefore, the results indicate that the indirect effect is statistically significant.

**Table 4:** Details of path analysis.

Path	Estimate	Std.all	Std. Err	95% CI	*p*
PE → USC (a1)	0.203	0.195	0.060	0.087 ~ 0.321	< 0.001
PE → AIB (c1)	0.683	0.511	0.108	0.498 ~ 0.922	< 0.001
USC → AIB (b1)	0.412	0.321	0.082	0.257 ~ 0.584	< 0.001

### 4.4 Moderation effect testing

**Figure 2:**
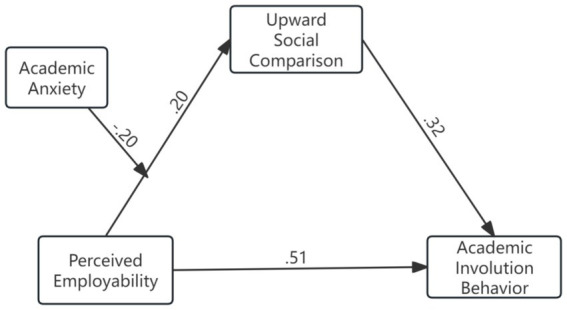
Structural equation model results corresponding to the hypothesized framework.

**Figure 3:**
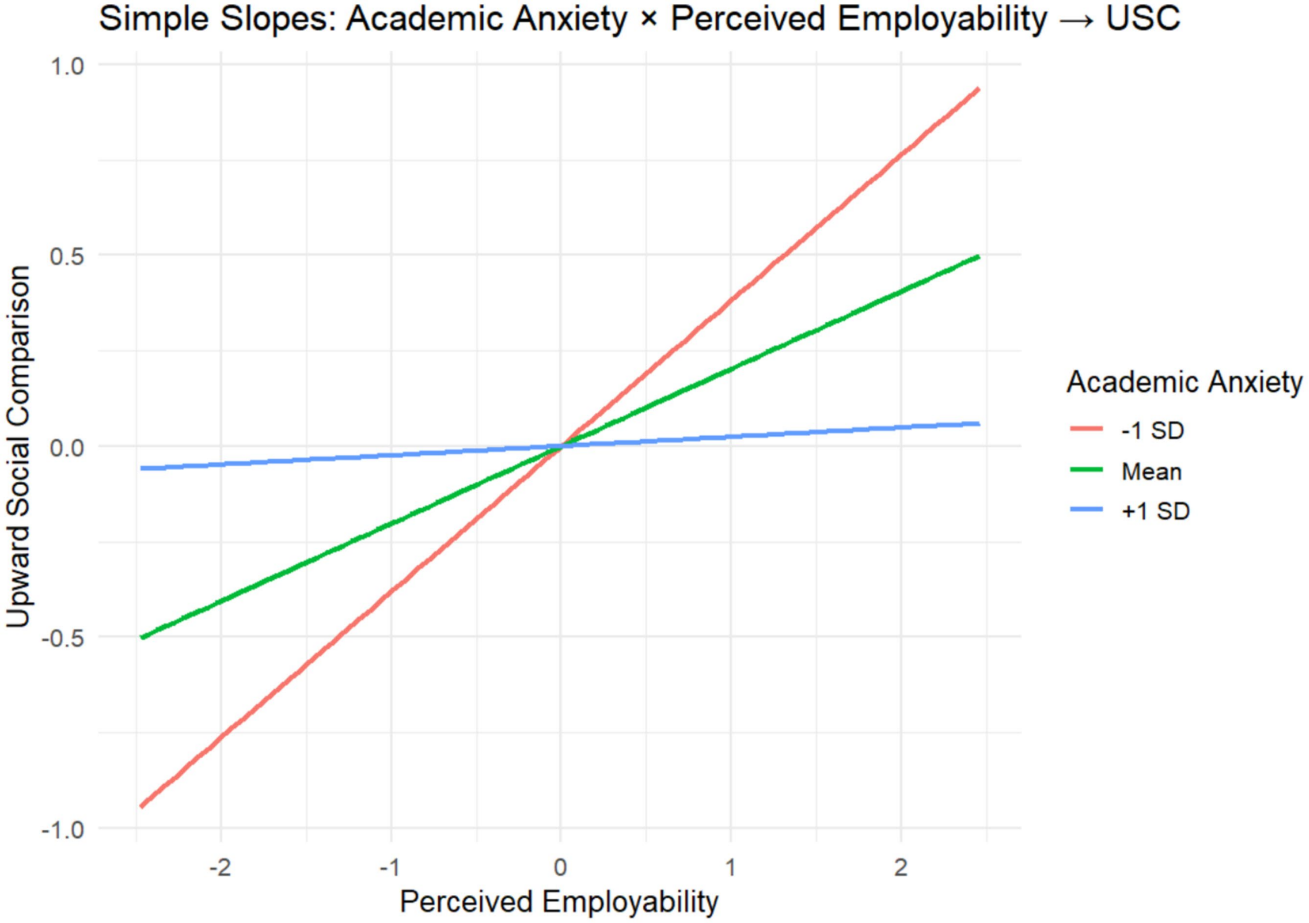
Simple slope analysis plot.

To further examine the moderating role of academic anxiety across different paths in the model, a fully moderated path model was constructed and estimated in R using lavaan 0.6–21. In addition to the main effects of perceived employability (PE), academic anxiety (AA), and upward social comparison (USC), the model incorporated two interaction terms: PE × AA and USC × AA. Maximum likelihood estimation (ML) was used, with robust (sandwich) standard errors, and the chi-square statistic was adjusted using the Yuan–Bentler correction to account for potential non-normality. The results indicated that the tested paths in the model were significantly moderated by academic anxiety (see Table 5). The simple slopes of the moderated path are presented in Figures 2, 3.

**Table 5:** Results of moderation effect test.

Path	Estimate	Std.all	Std. Err	95% CI	*p*
PE × AA → USC	−0.208	−0.199	0.054	−0.314 ~ −0.106	< 0.001

### 4.5 Common method bias test

This study employed Harman’s single-factor test. An unrotated factor analysis of all measurement items showed that the first factor explained 26.12% of the total variance, which is below the 40% threshold, indicating that there is no serious common method bias in this study. Furthermore, a single-factor confirmatory factor analysis (CFA) was conducted, loading all items onto a single latent factor. The results showed poor model fit (*χ*^2^/df = 9.458, RMR = 0.166, GFI = 0.557, RFI = 0.457, RMSEA = 0.130), indicating that all measurement items cannot be reasonably explained by a single factor. These results suggest that serious common method bias was not detected.

### 4.6 Multicollinearity test

To examine whether multicollinearity exists among the independent variables, this study used the Variance Inflation Factor (VIF). In SPSS, a regression analysis was conducted with academic involution behavior as the dependent variable and all other variables as independent variables. The VIF values were then examined, and all values were below 5 (Perceived Employability = 1.398, Upward Social Comparison = 1.097, Academic Anxiety = 1.373), indicating that serious multicollinearity was not present and that the variables could be safely included in the regression model (Akinwande et al., 2015).

### 4.7 Hypothesis testing

This study proposed four hypotheses. To test H1 through H3, structural equation modeling (SEM) was employed. The results indicated that three regression paths were statistically significant (*p* < 0.05), the 95% confidence intervals for all paths did not include zero, and all path coefficients were greater than 0.1. Therefore, hypotheses H1, H2, and H3 were supported. To test H4, which examined the moderating role of academic anxiety in the relationship between perceived employability and upward social comparison, a moderation analysis was conducted using lavaan 0.6–21 in RStudio. The results showed that this predictive path was statistically significant (*p* < 0.001, *|β|* > 0.1), confirming the presence of the moderating effect. Thus, hypothesis H4 was also supported.

## 5 Discussion

The JD-R model not only encompasses job demands and resources but also explicitly incorporates personal resources as important predictors of positive psychological states and performance (Xanthopoulou et al., 2007; Demerouti et al., 2001). Perceived employability (PE), as an individual’s subjective assessment of their prospects in the labor market, can be regarded as a personal resource that helps individuals cope with labor market uncertainties and influences their psychological and behavioral outcomes (De Cuyper et al., 2011; Vanhercke et al., 2016). Recent studies further link perceived employability to individual career behaviors, such as job search activities, supporting its application as a resource within the JD-R theoretical framework (Biramo et al., 2025).

Based on the empirical data collected in this study, perceived employability (PE) has a significant positive predictive effect on academic involution behavior (AIB) (H1; *β* = 0.511, *p* = 0.001), indicating that the initial hypothesis formulated from the model is largely confirmed. When an individual’s motivational processes are activated, they tend to actively pursue higher goals and seek self-validation through social comparison (Diel, 2021). In addition, the study found that perceived employability (PE) significantly positively predicts upward social comparison (USC) (H2; *β* = 0.195, *p* < 0.001). Individuals with higher perceived employability are more likely to focus on higher-achieving peers to evaluate their own advantages and gaps, which increases the tendency for upward social comparison. Similarly, upward social comparison (USC) has a significant positive predictive effect on academic involution behavior (AIB) (H3; *β* = 0.321, *p* < 0.001). According to the JD-R model’s motivational hypothesis, when individuals face high demands, if they can access sufficient resources (e.g., social support, sense of competence, perceived control), these resources are more effectively transformed into motivational energy, enhancing engagement and performance (Bakker et al., 2023). This empirical finding is also applicable to certain variables within the current model.

Academic involution is understood as a process in which individuals, under conditions of limited resources, are forced to compete with one another and continually expend themselves, ultimately leading to a sustained decline in the input-to-output ratio (Ling and Li, 2022). In the JD-R model, job demands refer to external or internal pressure factors that continuously consume an individual’s psychological or physical energy, compelling them to exert effort to maintain performance levels (Bakker and Demerouti, 2007). Therefore, in highly competitive academic and employment environments, students often assess their own abilities by comparing themselves with more capable peers, which in turn drives increased effort. Empirical data from this study indicate that perceived employability exerts a significant indirect effect on academic involution through upward social comparison (indirect *β* = 0.063, 95% CI [0.033–0.148]), suggesting that upward social comparison partially mediates the relationship. The higher the perceived employability, the more students believe in their ability to achieve goals, making them more likely to engage in proactive learning and self-improvement behaviors (Vanhercke et al., 2014). Compared with the path coefficient of academic involution behavior guided by upward social comparison (*β* = 0.321), the path coefficient guided directly by perceived employability (*β* = 0.511) is slightly higher, indicating that perceived employability more strongly drives academic involution than upward social comparison.

From the JD-R model perspective, perceived employability, as a core psychological resource, can stimulate students’ intrinsic motivation and sense of control toward career success, thereby encouraging greater effort in academics (Ma and Bennett, 2021). Upward social comparison, as a socially activated motivational mechanism triggered by perceived employability, exerts a more indirect influence rather than functioning as a mere comparison process (Eccles and Wigfield, 2002). This reflects that Chinese university students under a challenging employment situation tend to rely on self-improvement to cope with competition rather than solely adjusting behavior through social comparison. This finding confirms the dominant role of intrinsic motivation over extrinsic motivation in competitive learning behaviors (Deci and Ryan, 2000).

This study hypothesized that academic anxiety (AA) plays a moderating role in the relationship between perceived employability (PE) and upward social comparison (USC) behavior (H4). The results indicate that academic anxiety has a significant moderating effect on the predictive path from perceived employability to upward social comparison (*β* = −0.2, *p* < 0.001). Based on the survey data, we confirmed that due to the inherent characteristics of psychological resources, academic anxiety exerts a negative predictive effect along this path in the model. Specifically, as the level of academic anxiety increases, the positive predictive effect of perceived employability on upward social comparison weakens. This finding supports the core assumption of the health-impairment process in the JD-R model, which posits that excessive negative emotions erode an individual’s resource reserves, thereby weakening the positive effects of high perceived employability and potentially leading individuals to reduce upward social comparison behavior to avoid further psychological burden (Bakker and Demerouti, 2007).

From the perspective of Self-Regulation Theory, anxiety and stress undermine individuals’ self-efficacy and motivational orientation, resulting in psychological withdrawal (Bandura, 1997). This moderating effect also reveals the complex relationship between college students’ employment perceptions and social comparison behavior, challenging the linear assumption that higher perceived employability necessarily leads to increased upward social comparison. Even when students possess accurate employment awareness and capability assessment, if they are persistently affected by academic anxiety, their positive cognitions may not translate into constructive psychological behavior; instead, resource depletion caused by anxiety may occur. The remaining psychological resources may be insufficient to support the cognitive and emotional effort required for upward social comparison, ultimately preventing perceived employability from effectively promoting such comparisons and, in some cases, even prompting individuals to engage in downward social comparison to gain temporary psychological relief.

## 6 Conclusion

Based on the dual-pathway theory of the JD-R model, this study found that perceived employability (Job Resources) and academic demands (Job Demands) influence university students’ academic involution behavior through the motivational and health-impairment pathways, respectively. Perceived employability, as a positive psychological resource, can activate students’ motivation and self-efficacy, thereby promoting proactive engagement. In contrast, high academic demands and social comparison pressures consume psychological energy and induce anxiety, leading to passive involution behavior.

Empirical results indicate that perceived employability significantly positively predicts academic involution behavior and upward social comparison, while upward social comparison also significantly positively predicts academic involution behavior. This suggests that when students perceive themselves as having higher employability competitiveness, they tend to invest more effort in academic tasks to maintain or strengthen this advantage. These findings support the motivational process in the JD-R model, in which personal resources activate individuals’ proactive engagement.

Furthermore, the study found that upward social comparison partially mediates the relationship between perceived employability and academic involution behavior. High perceived employability leads students to assess their performance and future development by comparing themselves with higher-achieving peers, reflecting resource-driven social comparison motivation. Within the JD-R framework, social comparison can be viewed as an “internalized job demand,” which has become a key factor driving students’ excessive competitive engagement.

The study also revealed that academic anxiety negatively moderates the predictive path from perceived employability to upward social comparison. High levels of academic anxiety weaken the promoting effect of perceived employability on upward social comparison, causing students’ social comparison behavior to become more defensive and reducing their tendency for proactive comparison. This finding highlights the boundary role of emotional stress factors in the JD-R model’s health-impairment process: while perceived employability can encourage students to learn from higher-achieving peers, prolonged high academic anxiety can diminish this positive effect.

### 6.1 Policy implications

Based on the empirical findings of this study, perceived employability was found to significantly and positively predict academic involution behavior among Chinese university students. This indicates that in the current competitive employment environment, overly high perceptions of employability may trigger excessive academic engagement and anxiety. To alleviate academic and psychological pressures, especially among students approaching graduation, it is recommended that policy and educational practices focus on fostering a balanced development of students’ rational understanding of the employment landscape and their self-efficacy. Universities should incorporate differentiated career planning guidance and counseling courses into career development education, helping students accurately assess their abilities and career goals, thereby avoiding “involution” caused by unrealistic employment expectations.

This study found that upward social comparison mediates the relationship between perceived employability and academic involution behavior. In contexts of high perceived employability, students are more likely to compare themselves with high-achieving peers to motivate their learning, but this process may also induce excessive competition and psychological exhaustion. Therefore, policymakers and university administrators should promote a cooperative and constructive competitive culture on campus, guiding students to use social comparison as a means for self-growth and skill development rather than anxiety-driven competition. Additionally, mental health education and emotional regulation training should be continuously strengthened, with psychological counseling services and stress-management courses provided to enhance students’ emotional adjustment abilities.

## 7 Limitations

Several limitations remain in this study. First, the cross-sectional survey design makes it difficult to establish causal relationships between variables; future research could employ longitudinal tracking or experimental designs to further validate the findings. Second, the data were collected through self-reported questionnaires, which may be subject to common method bias. Although anonymity and statistical controls were applied, future studies should incorporate multi-source data to improve reliability. Finally, future research could test the robustness and generalizability of the model across broader samples and variable frameworks.

## Data Availability

The original contributions presented in the study are included in the article/Supplementary material, further inquiries can be directed to the corresponding author.

## Footnotes

1
https://www.credamo.com
