# Supplementary material for: The influence mechanism of academic involution behavior among Chinese college students: a moderated mediation analysis based on the JD-R model
Source: Front Psychol. 2026 Mar 6;17:1729314. doi: 10.3389/fpsyg.2026.1729314 (PMC13003459; doi:10.3389/fpsyg.2026.1729314)
Supplement: Supplementary file 1 [file Supplementary_file_1.docx]

**Appendix 1. Item-Level Factor Structure and Item Deletion Decisions**

| **Scale & Items** | **Primary**  **Factor** | **Processing results** |
| --- | --- | --- |
| **Appendix A. Employability scale items** |  |  |
| 1a. I achieve high grades in relation to my studies | .317 | Factor loadings are too low, so delete. |
| 1b. I regard my academic work as top priority |  |  |
| 2a. Employers are eager to employ graduates from my university |  |  |
| 2b. The status of this university is a significant asset to me in job seeking |  | Preserve results |
| 3a. Employers specifically target this university in order to recruit individuals from my subject area(s) |  |  |
| 3b. My university has an outstanding reputation in my field(s) of study |  | Preserve results |
| 4a. A lot more people apply for my degree than there are places available | .412 | Factor loadings are too low, so delete. |
| 4b. My chosen subject(s) rank(s) highly in terms of social status |  |  |
| 5a. People in the career I am aiming for are in high demand in the external labour market |  | Preserve results |
| 5b. My degree is seen as leading to a specific career that is generally perceived as highly desirable |  |  |
| 6a. There is generally a strong demand for graduates at the present time |  | Preserve results |
| 6b. There are plenty of job vacancies in the geographical area where I am looking |  |  |
| 7a. I can easily find out about opportunities in my chosen field |  |  |
| 7b. The skills and abilities that I possess are what employers are looking for |  | Preserve results |
| 8a. I am generally confident of success in job Interviews and selection events |  | Preserve results |
| 8b. I feel I could get any job so long as my skills and experience are reasonably relevant |  |  |
|  |  |  |
| **Chinese College Students’ Academic Involution Scale** |  |  |
| 1. I will attend a tutorial class privately to improve myself so as not to be left behind by others |  | Preserve results |
| 2. I will follow some knowledge‐sharing social media accounts (such as bilibili accounts, microblog accounts, or Xiaohongshu, etc.) to improve myself, so as to avoid being left behind by others |  | Preserve results |
| 3. To get better results, I will consult with the senior students about the relevant knowledge of the courses I have registered (such as the past exam questions, test materials, and teacher's notes |  | Preserve results |
| 4. To achieve excellent results on the final exam, I will purchase some learning resources (such as PPT slides, reference books, past exam questions, and so forth) |  | Preserve results |
| 5. I will often inquire about my classmates' learning situation, and if I feel that the quality of my homework is not as good as theirs, I will modify it to avoid being left behind by others | .296 | Factor loadings are too low, so delete. |
| 6. I would go to the library on weekends and other breaks so as not to be left behind |  | Preserve results |
| 7. I get up early and come back late to the dormitory every day to study so as not to be left behind |  | Preserve results |
|  |  |  |
| **Directional-Upward Comparison Subscale** |  |  |
| 1. When it comes to my personal life, I sometimes compare myself with others who have it better than I do. |  | Preserve results |
| 1. When I consider how I am doing socially (e.g., social skills, popularity), I prefer to compare with others who are more socially skilled than I am. |  | Preserve results |
| 1. When evaluating my current performance (e.g., how I am doing at home, work, school, or wherever), I often compare with others who are doing better than I am. |  | Preserve results |
| 1. When I wonder how good I am at something, I sometimes compare myself with others who are better at it than I am. |  | Preserve results |
| 1. When things are going poorly, I think of others who have it better than I do. |  | Preserve results |
| 1. I sometimes compare myself with others who have accomplished more in life than I have. |  | Preserve results |
|  |  |  |
| **Academic Anxiety Scale** |  |  |
| 1. I often worry that my best is not as good as expected in school. |  | Preserve results |
| 1. I tend to put off doing school work because it stresses me. | .383 | Factor loadings are too low, so delete. |
| 1. I often worry that I am not doing assignments properly. |  | Preserve results |
| 1. I am less confident about school than my classmates. |  | Preserve results |
| 1. I have a sense of dread when I am in my classrooms. |  | Preserve results |
| 1. I tend to find my instructors intimidating. |  | Preserve results |
| 1. I spend much of my time at school worrying about what is next. |  | Preserve results |
| 1. There is something about school that scares me. |  | Preserve results |
| 1. I’m concerned about what my classmates think about my abilities. |  | Preserve results |
| 1. I often feel sick when I need to work on a major class assignment. |  | Preserve results |
| 1. I have a hard time handling school responsibilities |  | Preserve results |
