# Supplementary material for: The influence mechanism of academic involution behavior among Chinese college students: a moderated mediation analysis based on the JD-R model
Source: Front Psychol. 2026 Mar 6;17:1729314. doi: 10.3389/fpsyg.2026.1729314 (PMC13003459; doi:10.3389/fpsyg.2026.1729314)
Supplement: Supplementary file 2 [file Supplementary_file_2.docx]

**Appendix 2. Original Scales and Full Item Sets**

**Employability scale items**

**Appendix A. Employability scale items**

1a. I achieve high grades in relation to my studies

1b. I regard my academic work as top priority

2a. Employers are eager to employ graduates from my university

2b. The status of this university is a significant asset to me in job seeking

3a. Employers specifically target this university in order to recruit individuals from my subject area(s)

3b. My university has an outstanding reputation in my field(s) of study

4a. A lot more people apply for my degree than there are places available

4b. My chosen subject(s) rank(s) highly in terms of social status

5a. People in the career I am aiming for are in high demand in the external labour market

5b. My degree is seen as leading to a specific career that is generally perceived as highly desirable

6a. There is generally a strong demand for graduates at the present time

6b. There are plenty of job vacancies in the geographical area where I am looking

7a. I can easily find out about opportunities in my chosen field

7b. The skills and abilities that I possess are what employers are looking for

8a. I am generally confident of success in job Interviews and selection events

8b. I feel I could get any job so long as my skills and experience are reasonably relevant

**Appendix B. Ambition scale items**

A1. I want to be in a position to do mostly work which I really like

A2. I am satisfied with the progress I have made meeting my goals for the development of new skills

A3. I have clear goals for what I want to achieve in life

A4. I regard myself as highly ambitious

A5. I feel it is urgent that I get on with my career development

A6. What I do in the future is not really important (reverse-scored)

**Appendix C. University commitment scale items**

UC1. I talk up this university to my friends as a great university to be at

UC2. (I would have accepted almost any type of course offer in order to come to this university1)

UC3. I find that my values and this university’s values are very similar

UC4. I am proud to tell others that I am at this university

UC5. Being at this university really inspires the best in me in the way of study performance

UC6. I am extremely glad I chose this university over others I was considering at the time I joined

UC7. I really care about this university and its future

UC8. For me this is the best of all universities to be a member of

**F1 Academic Behavior (AB)**

1. I will attend a tutorial class privately to improve myself so as not to be left behind by others

2. I will follow some knowledge‐sharing social media accounts (such as bilibili accounts, microblog accounts, or Xiaohongshu, etc.) to improve myself, so as to avoid being left behind by others

3. To get better results, I will consult with the senior students about the relevant knowledge of the courses I have registered (such as the past exam questions, test materials, and teacher's notes

4. To achieve excellent results on the final exam, I will purchase some learning resources (such as PPT slides, reference books, past exam questions, and so forth)

5. I will often inquire about my classmates' learning situation, and if I feel that the quality of my homework is not as good as theirs, I will modify it to avoid being left behind by others

6. I would go to the library on weekends and other breaks so as not to be left behind

7. I get up early and come back late to the dormitory every day to study so as not to be left behind

**F2 Social Activity (SA)**

1. I don't like it very much, but I will participate in various competitions so that my comprehensive evaluation results will not be left behind by others

2. Although I don't like it very much, I will join various clubs so that my comprehensive evaluation results will not be left behind by others

3. Although I don't like it very much, I will take part in various voluntary activities so that my comprehensive evaluation results will not be left behind by others

4. Although I don't like it very much, I will attend various lectures so that my comprehensive evaluation results will not be left behind by others

5. Although I don't like it very much, I will participate in social practice in winter and summer vacation so that my comprehensive evaluation results will not be left behind by others

**F3 Social Interaction (SI)**

1. I will actively help my roommates to avoid losing in various evaluations

2. I will keep a good relationship with my classmates to avoid losing in various evaluations

3. I will actively interact with teachers and strive to achieve no lower grades than others

4. I will actively reply to the tutor's comments to avoid being defeated in various evaluations

**Comparison Subscale**

**Non-directional Comparison Subscale**

1. I often compare myself with others with respect to what I have accomplished in life.

2. If I want to learn more about something I try to find out what others think about it.

3. I always pay a lot of attention to how I do things compared with how others do things.

4. I often compare how my loved ones (boy or girlfriend, family members, etc.) are doing

with how others are doing.

5. I always like to know what others in a similar situation would do.

6. I am not the type of person who compares often with others. (Reverse score)

7. If I want to find out how well I’ve done something, I compare what I have done with

how others have done.

8. I often try to find out what others think who face similar problems as I face.

9. I often like to talk with others about mutual opinions and experiences.

10. I never consider my situation in life relative to that of other people. (Reverse score)

11. I often compare how I am doing socially (e.g., social skills, popularity) with other

people.

**Directional-Upward Comparison Subscale**

12. When it comes to my personal life, I sometimes compare myself with others who have it

better than I do.

13. When I consider how I am doing socially (e.g., social skills, popularity), I prefer to

compare with others who are more socially skilled than I am.

14. When evaluating my current performance (e.g., how I am doing at home, work, school,

or wherever), I often compare with others who are doing better than I am.

15. When I wonder how good I am at something, I sometimes compare myself with others

who are better at it than I am.

16. When things are going poorly, I think of others who have it better than I do.

17. I sometimes compare myself with others who have accomplished more in life than I

have.

**Directional-Downward Comparison Subscale**

18. When it comes to my personal life, I sometimes compare myself with others who have it

worse than I do.

19. When I consider how I am doing socially (e.g., social skills, popularity), I prefer to

compare with others who are less socially skilled than I am.

20. When evaluating my current performance (e.g., how I am doing at home, work, school,

or wherever), I often compare with others who are doing worse than I am.

21. When I wonder how good I am at something, I sometimes compare myself with others

who are worse at it than I am.

22. When things are going poorly, I think of others who have it worse than I do.

23. I sometimes compare myself with others who have accomplished less in life than I have.

**Academic Anxiety Scale**

1. I often worry that my best is not as good as expected in school.
2. I tend to put off doing school work because it stresses me.
3. I often worry that I am not doing assignments properly.
4. I am less confident about school than my classmates.
5. I have a sense of dread when I am in my classrooms.
6. I tend to find my instructors intimidating.
7. I spend much of my time at school worrying about what is next.
8. There is something about school that scares me.
9. I’m concerned about what my classmates think about my abilities.
10. I often feel sick when I need to work on a major class assignment.
11. I have a hard time handling school responsibilities
